# Comparative Efficacy of the Generalized Anxiety Disorder 7-Item Scale and the Edinburgh Postnatal Depression Scale as Screening Tools for Generalized Anxiety Disorder in Pregnancy and the Postpartum Period

**DOI:** 10.1177/070674371405900806

**Published:** 2014-08

**Authors:** William Simpson, Melanie Glazer, Natalie Michalski, Meir Steiner, Benicio N Frey

**Affiliations:** 1 Student, MiNDS Neuroscience Program, McMaster University, Hamilton, Ontario.; 2 Student, Department of Psychology, Neuroscience and Behaviour, McMaster University, Hamilton, Ontario.; 3 Professor Emeritus, Department of Psychiatry and Behavioural Neurosciences, McMaster University, Hamilton, Ontario; Psychiatrist, Women’s Health Concerns Clinic, St Joseph’s Healthcare, Hamilton, Ontario.; 4 Associate Professor, Department of Psychiatry and Behavioural Neurosciences, McMaster University, Hamilton, Ontario; Director, Women’s Health Concerns Clinic, St Joseph’s Healthcare, Hamilton, Ontario; Academic Head, Mood Disorders Program, St Joseph’s Healthcare, Hamilton, Ontario.

**Keywords:** pregnancy, postpartum, anxiety, Edinburgh Postnatal Depression Scale, Generalized Anxiety Disorder 7-item Scale

## Abstract

**Objective:**

About 24.1% of pregnant women suffer from at least 1 anxiety disorder, 8.5% of whom suffer specifically from generalized anxiety disorder (GAD). GAD is often associated with major depressive disorder (MDD). During the perinatal period, the presence of physical and somatic symptoms often makes differentiation between depression and anxiety more challenging. To date, no screening tools have been developed to detect GAD in the perinatal population. We investigated the psychometric properties of the GAD 7-item Scale (GAD-7) as a screening tool for GAD in pregnant and postpartum women.

**Methods:**

Two hundred and forty perinatal women (*n* = 155 pregnant and *n* = 85 postpartum) referred for psychiatric consultation were enrolled. On the day of initial assessment, all women completed the GAD-7 and the Edinburgh Postnatal Depression Scale (EPDS). Diagnostic and Statistical Manual of Mental Disorders, Fourth Edition–based diagnoses were made by experienced psychiatrists. Scores from the GAD-7 and EPDS were compared with the clinical diagnoses to evaluate the psychometric properties of the GAD-7 and EPDS when used as a screening tool for GAD.

**Results:**

The GAD-7 yielded a sensitivity of 61.3% and specificity of 72.7% at an optimal cut-off score of 13. Compared with the EPDS and the EPDS-3A subscale, the GAD-7 displayed greater accuracy and specificity over a greater range of cut-off scores and more accurately identified GAD in patients with comorbid MDD.

**Conclusion:**

Our findings suggest that the GAD-7 represents a clinically useful scale for the detection of GAD in perinatal women.

## Abstract (fr)

**Objectif:**

Environ 24,1 % des femmes enceintes souffrent d’au moins 1 trouble anxieux, et 8,5 % d’entre elles souffrent spécifiquement du trouble anxieux généralisé (TAG). Le TAG est souvent associé au trouble dépressif majeur (TDM). Durant la période périnatale, la présence de symptômes physiques et somatiques rend la différenciation entre dépression et anxiété encore plus difficile. Jusqu’ici, aucun instrument de dépistage n’a été mis au point pour détecter le TAG dans la population périnatale. Nous avons recherché les propriétés psychométriques de l’échelle à 7 items du TAG (TAG-7) comme instrument de dépistage du TAG chez les femmes enceintes et postpartum.

**Méthodes:**

Deux cent quarante femmes périnatales (*n* = 155 enceintes et *n* = 85 postpartum) adressées à une consultation psychiatrique ont été inscrites. Le jour de l’évaluation initiale, toutes les femmes ont répondu à la TAG-7 et à l’échelle de dépression postnatale d’Édimbourg (EDPE). Des diagnostics basés sur le Manuel diagnostique et statistique des troubles mentaux, 4^e^ édition, ont été posés par des psychiatres expérimentés. Les scores à la TAG-7 et à l’EDPE ont été comparés avec les diagnostics cliniques pour évaluer les propriétés psychométriques de la TAG-7 et de l’EDPE lorsque ces échelles servent d’instrument de dépistage du TAG.

**Résultats:**

La TAG-7 a produit une sensibilité de 61,3 % et une spécificité de 72,7 % au seuil d’inclusion optimal de 13. Comparé à l’EDPE et à la sous-échelle EDPE-3A, la TAG-7 présentait une précision et une spécificité plus grandes sur une étendue plus longue de seuils d’inclusion, et identifiait plus exactement le TAG chez les patientes souffrant de TDM comorbide.

**Conclusion:**

Nos résultats suggèrent que la TAG-7 représente une échelle cliniquement utile pour la détection du TAG chez les femmes périnatales.

Anxiety disorders as a group are one of the most prevalent psychiatric conditions with an estimated lifetime prevalence of 28.8% in the general population.^1^ GAD specifically is characterized by a 6-month period of excessive, uncontrollable worry regarding life events or activities, accompanied by at least 3 symptoms of negative affect or tension. GAD has a lifetime prevalence of 5.7%^1^ and if left untreated can become a chronic disorder with low rates of remission.^2^,^3^ Risk factors of GAD include family history, an increase in situational stressors and (or) stressful life events,^4^,^5^ and a history of physical or emotional trauma.^3^,^6^ Many people with GAD report heightened anxiety beginning in childhood,^4^ with clinically significant anxiety symptoms emerging during the late teens through to the late twenties.^7^

Significant sex differences exist in the prevalence of GAD. Studies of both lifetime and 12-month prevalence indicate that women are twice as likely to suffer from GAD, compared with men.^8^–^12^ Risk for GAD also appears to increase during the perinatal period. Prevalence studies suggest that about 8.5% of women suffer from GAD during pregnancy,^13^ while rates of 4.4% to 8.2% have been reported in the postpartum period.^14^–^16^ These prevalence rates are considerably higher than those observed in the general population (3.1%) during a 12-month period.^17^

During pregnancy, women typically experience an increase in worries relating to the health of their baby, their own health, financial matters, childcare, and parenting.^18^ They also often experience pregnancy-related increases in physical and somatic symptoms, such as fatigue, muscle tension, poor concentration, sleep difficulties, irritability, and restlessness. This may lead physicians to overlook a clinical diagnosis of GAD, assuming that these symptoms are simply related to pregnancy itself. GAD is also a highly comorbid disorder, particularly with MDD. Numerous studies have reported high comorbidity rates, varying between 15% and 69%.^18^–^22^ The presence of one or more psychiatric comorbidities is known to increase the severity of symptoms and decrease the likelihood of remission.^23^–^25^ This frequent comorbidity, coupled with a significant overlap in core symptomatology and a perinatal increase in anxious and somatic symptoms, can often make correctly diagnosing GAD during the perinatal period challenging.

**Clinical Implications**

- The GAD-7 is a clinically meaningful instrument when assessing perinatal anxiety.
- Use of the GAD-7 will aid in differentiating clinically significant anxiety from normal increases in pregnancy-related anxiety.

**Limitations**

- Clinical diagnostic interviews were used rather than standardized semi-structured interviews (for example, the Structured Clinical Interview for DSM Disorders).
- Pregnancy-related increases in anxiety and somatic symptoms may have masked some of the GAD-7 specificity.

Heightened maternal anxiety during pregnancy has been associated with long-term consequences for the unborn fetus. Some studies indicate that children of anxious mothers are more likely to have low birth weights, be born prematurely, and be at greater risk for future cognitive and behavioural difficulties, such as attention deficit and aggressive disorders.^6^,^18^,^26^ However, it is important to note that some maternal anxiety may be beneficial and can increase the rate of neonatal neural development.^27^

There are many existing scales used for the screening of various anxiety disorders before and after pregnancy (Table 1). The EPDS, originally developed to screen for depression, has been used as a self-rated screening tool for perinatal anxiety disorders, including GAD.^28^ Grigoriadis et al^29^ examined the performance of the EPDS for the screening of GAD in women referred for perinatal psychiatric assessment. The EPDS achieved a sensitivity of 70%, specificity of 82%, PPV of 79%, and NPV of 74% at a cut-off score of 12.^29^ The EPDS can be further divided into anxiety (items 3, 4, 5; EPDS-3A) and depression (items 1, 2, 6–10) subscales.^30^,^31^ Grigoriadis et al also examined the use of the EPDS-3A (cut-off > 4, range 0 to 9) to screen for GAD. Using this subscale resulted in higher sensitivity (88%, compared with 70%) but significantly lower specificity (49%, compared with 82%), compared with the full EPDS.^29^ Matthey30 also examined the use of the EPDS-3A to screen for GAD and PD in a sample of postpartum women from the general population. A cutoff score of 6 yielded a sensitivity of 66.7%, specificity of 88.2%, PPV of 31.6%, and NPV of 97.0%.^30^ Together, these results suggest that, while the EPDS and EPDS-3A subscale may be adequate, neither is an optimal screening tool for perinatal GAD.

The GAD-7 is a self-rated assessment developed by Spitzer et al^32^ to screen for GAD in primary care populations. During its initial validation using a cut-off score of 10, the GAD-7 yielded a sensitivity of 89%, specificity of 82%, PPV of 29%, and NPV of 99% in a primary care sample.^32^ Further studies of the GAD-7 in the general population have yielded a sensitivity of 60.6% and a specificity of 87.6% at a cut-off score of 10.^33^ Examination of the GAD-7 in psychiatric populations is limited, though a recent study^34^ observed a sensitivity of 64% and a specificity of 57% at an optimal cut-off score of 13 in psychiatric patients referred to an acute partial hospital program.

While the GAD-7 has proven to be a useful screening tool for GAD in the primary care population, its use as a screening tool for GAD in pregnant and postpartum women has not been assessed. In our study, we compared the efficacy of the GAD-7, EPDS, and EPDS-3A as screening tools for GAD in a sample of perinatal women.

## Methods

Our study was approved by the McMaster Integrated Research Ethics Board. Two hundred and forty women (*n* = 155 pregnant and *n* = 85 postpartum) referred for psychiatric consultation at the WHCC at St Joseph’s Healthcare Hamilton between January 2011 and February 2013 were assessed through retrospective chart review. Most were referred to the WHCC by family doctors and obstetric and midwifery clinics in Hamilton, Ontario. On the day of initial assessment, all women completed the GAD-7 and the EPDS.^28^ The DSM-IV–based diagnoses were made by experienced psychiatrists. Total scores from both scales, together with psychiatric diagnoses and demographic information, were extracted for each patient. Scores from the GAD-7 were compared with the clinical diagnoses to evaluate the psychometric measures of the GAD-7 when used as a screening tool for GAD. To assess how the GAD-7 performed relative to other previously validated perinatal anxiety screening tools, we computed the psychometric properties of the EPDS and the EPDS-3A subscale. Given the high comorbidity between GAD and MDD we also examined whether the GAD-7, EPDS, and EPDS-3A were effective at identifying GAD in patients with comorbid MDD and GAD.

Sensitivity, specificity, PPV, NPV, and chance-corrected level of agreement (kappa) were calculated using the statistical package R (version 2.13; Vienna, Austria, 2014). Receiver operator characteristic curves and AUC estimates were also computed using R. Patients with a “rule out,” “possible,” or “query” diagnoses of GAD at the initial assessment were considered unaffected. Psychometric data were interpreted according to the criteria developed by Blacker and Endicott^35^ (more than 0.80 = excellent or highly correlated; 0.80 to 0.70 = good or adequately correlated; 0.69 to 0.50 = fair or fairly correlated; and less than 0.50 = poor or poorly correlated).

## Results

Demographic information is presented in Table 2. The age range of the sample was 16 to 46 years, with a mean age of 30.5 years (SD 5.7). Most of the women were pregnant (*n* = 155, 64.6%), while the remainder were postpartum (*n* = 85, 35.4%). Most women were married (*n* = 153, 63.8%), had received a bachelor’s degree or higher (*n* = 62, 25.8%), and had a history of psychiatric disorders in their immediate family (*n* = 164, 68.3%). MDD was the most prevalent primary psychiatric diagnosis (*n* = 108, 45.0%), followed by GAD (*n* = 35, 14.6%). Consistent with previous studies, in our sample a significant proportion of people with MDD had comorbid GAD (45/108, 41.6%).

### Generalized Anxiety Disorder 7-Item Scale

**Figure 1:**
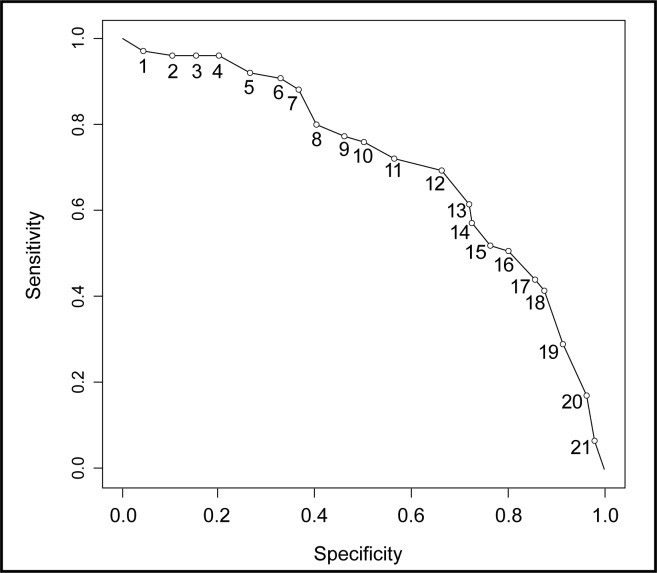
**Sensitivity and specificity of the Generalized Anxiety Disorder 7-item Scale for the detection of generalized anxiety disorder at varying cut-off scores**

Using the previously established cut-off score of 10,^32^ the GAD-7 yielded good sensitivity (76.0%), poor specificity (51.5%), poor PPV (41.6%), good NPV (82.5%), and poor kappa (0.22) as a screening for GAD. A cut-off score of 13 yielded the best fitting model, with a sensitivity of 61.3% and specificity of 72.7%, with a PPV of 50.5%, NPV of 80.5%, and kappa of 0.32 (Figure 1). The psychometric properties of the GAD-7 did not improve when patients presenting with a provisional clinical diagnosis of GAD (that is, “query,” “rule-out,” and “possible”) were considered to be positive for GAD. Similarly, requiring an answer of “very difficult” or “extremely difficult” on the GAD-7 supplementary question (“If you checked off any problems, how difficult have these made it for you to do your work, take care of things at home, or get along with other people?”) did not improve the psychometric properties of the scale.

### Edinburgh Postnatal Depression Scale and Edinburgh Postnatal Depression Scale–Anxiety Subscale

EPDS cut-off scores ranging from 10 to 13 have been used in previous studies to screen for MDD in perinatal samples.^36^,^37^ EPDS scores were significantly correlated with scores on the GAD-7 (*r* = 0.70, *P* < 0.001). Using the above cut-off scores to screen for GAD, the EPDS achieved excellent sensitivity (77.3% to 89.3%) and NPV (79.2% to 84.4%), but poor specificity (26.7% to 40.3%) and PPV (36.2% to 37.6%). Kappa values were very low, ranging from 0.12 to 0.14. Using the EPDS-3A subscale to screen for GAD in our sample yielded excellent NPV (81.1%), fair sensitivity (68.0%) and specificity (63.5%), poor PPV (46.3%), and a kappa (0.28) at an optimal cut-off score of 7.

### Comorbid Generalized Anxiety Disorder and Major Depressive Disorder

Given the high rates of comorbidity between MDD and GAD in our sample (41.6%), we examined the performance of the GAD-7, EPDS, and EPDS-3A at detecting GAD in people with a comorbid diagnosis of MDD. At an optimal cut-off score of 13, the GAD-7 produced fair sensitivity (66.7%), specificity (68.7%), poor PPV (32.9%), excellent NPV (89.9%), and a kappa of 0.25. The EPDS produced similar results, with fair sensitivity (66.7%), specificity (65.4%), poor PPV (31.2%), excellent NPV (89.2%), and a kappa of 0.22 at a cut-off score of 17. Psychometric properties of the EPDS-3A were similar to those of the EPDS, with fair sensitivity (68.9%), specificity (58.8%), poor PPV (28.1%), excellent NPV (88.9%), and a kappa of 0.18 at a cut-off score of 7.

### Area Under the Curve Analysis

AUC was calculated to examine the performance of each screening tool in detecting GAD alone or GAD in the presence of comorbid MDD. Accuracy of the screening tool was interpreted as low (AUC = 0.50 to 0.70), moderate (AUC = 0.70 to 0.90), or high (AUC > 0.90).^38^ AUC analyses showed that accuracy of the GAD-7 was moderate for detecting GAD and GAD in the presence of comorbid MDD (0.71 and 0.74, respectively). The EPDS was less accurate in detecting both GAD alone (0.62) and GAD with comorbid MDD (0.68). The EPDS-3A was slightly more accurate than the EPDS (GAD alone, 0.69; GAD and MDD, 0.67).

## Discussion

Our study is the first to analyze the sensitivity and specificity of the GAD-7 in pregnant and postpartum women. Our results indicate that the psychometric properties of the GAD-7 are slightly better than those of the EPDS and EPDS-3A for the detection of GAD in this population. Modifying the scoring algorithm did not result in an improvement in the psychometric properties. When used to screen for GAD, the GAD-7 displayed higher specificity over a greater range of cut-off scores than the EPDS or EPDS-3A. Both the AUC and kappa values for the GAD-7 also exceeded those of the EPDS and EPDS-3A, indicating greater overall accuracy of the instrument. Further, this increased specificity, higher AUC, and higher kappa values were maintained when the GAD-7 was used to identify GAD in patients with comorbid MDD. These results also indicate that a higher score on the GAD-7 is more specific to symptoms of GAD than a higher score on the EPDS or EPDS-3A.

In our study, the sensitivity and specificity of the GAD-7 were comparable with values obtained in other study populations, albeit at higher cut-off scores. This discrepancy in cut-off score could be due to several factors. The cut-off score for any screening tool is largely dependent on prevalence of the indexing condition in the study population. In the general population, the prevalence of GAD symptoms is relatively low, thus a lower symptom count (that is, cut-off score) is required to distinguish disorder from nondisorder. However, in a population referred for psychiatric assessment, the probability that GAD symptoms will be present is markedly higher. Thus, to obtain similar psychometric properties, the cut-off score is expected to be higher.^39^

The use of self-rated instruments for the detection of perinatal anxiety disorders was recently reviewed by Meades and Ayers.^40^ In their review, the researcher highlight the use of the HADS, the STAI, the K-10, and the GHQ-28 for their use in various pregnant and postpartum populations. While many of these scales display superior psychometric properties to those obtained in our sample, the authors note that several issues prevent these scales from being accurate perinatal screening tools. The HADS and STAI both contain items where the ratings may be confounded by symptoms of normal pregnancy (for example, HADS: “I can sit at ease and feel relaxed”; STAI: “I tire quickly” and “I feel rested”), potentially increasing the incidence for false positives. The K-10 has been used to screen for social anxiety disorder and PD, but has not yet been validated to assess perinatal GAD. The GHQ-28 has shown some promise for the detection of perinatal anxiety. However, it remains unclear which of the 4 scoring methods for the GHQ-28 provide the most accurate diagnosis in this population.^40^ In a recent study^41^ comparing the performance of the EPDS-3A, HADS-A, Pregnancy-Related Thoughts, Pregnancy-Related Anxiety Questionnaire–Revised, and the MGMQ scales in pregnant women, the MGMQ was considered superior for detecting women meeting diagnostic criteria for an anxiety disorder according to the Mini-International Neuropsychiatric Interview. Unfortunately, the psychometric properties of these self-reported scales were not reported in the manuscript.

In our sample, the psychometric properties of the EPDS and EPDS-3A were poorer than those obtained from similar populations.^29^,^30^ This may be accounted for in that we have compared the performance of these self-reported scales against clinical psychiatric diagnoses made by psychiatrists with extensive experience in women’s mental health, in contrast to semi-structured diagnostic interviews (for example, Structured Clinical Interview for DSM Disorders) administered by trained students and research assistants.^29^,^30^ As the central question is whether these scales are useful in clinical settings, diagnoses made by experienced clinical psychiatrists and mental health providers may serve as a more accurate comparison. It is also possible that pregnancy-related increases in both anxiety and somatic symptoms, coupled with a higher degree of psychiatric symptoms and comorbid conditions in our population, may have contributed to the lower diagnostic specificity and poorer psychometric properties.

In conclusion, our results highlight that the GAD-7 represents a clinically meaningful instrument when screening for GAD in a perinatal population. Our findings indicate that a high score on the GAD-7 is more specific to GAD than a high score on the EPDS or EPDS-3A. The GAD-7 also maintains its clinical utility when used to assess GAD in the presence of comorbid MDD. Undiagnosed and untreated GAD can result in long-lasting negative outcomes for mothers and their children. While better screening questionnaires for GAD and anxiety in perinatal women are awaited, the GAD-7 represents an easily administered tool that can aid clinicians in establishing a diagnosis of GAD in perinatal populations. Continuous research on the development of new screening tools for GAD, with superior psychometric properties specifically validated for pregnant and postpartum women, is encouraged.

## Figures and Tables

**Table 1:** Sensitivity and specificity of self-report screening tools for various anxiety disorders in general and perinatal populations

Screening tool	Disorder	General population			Perinatal population		
		Sensitivity, %	Specificity, %	Cut-off	Sensitivity, %	Specificity, %	Cut-off
EPDS^29^	GAD	—	—	—	70	82	>12
EPDS-3A^29^	GAD	—	—	—	88	49	>4
HADS^40^,^42^	Anxiety	87.5	90.6	8	92.9	90.2	8
STAI^40^	Anxiety	—	—	—	80.95	79.75	>40
K-10^40^,^43^	Any anxiety disorder	79	76	20	—	—	—
	GAD	94	67	20	—	—	—
	Panic disorder	81	70	20	50	98	—
	Social phobia	78	68	20	100	98	—
	PTSD	—	—	—	50	80	—
GHQ-28^a,40,44^	Anxiety, normal GHQ scoring	79.7	79.2	—	75	83	3
	Anxiety, C-GHQ scoring	—	—	—	82	85	7

a Two scoring methods were analyzed for the GHQ-28 scale, including normal GHQ scoring (rating each item on a bimodal scale 0-0 to 1-1) and C-GHQ scoring (using a rating of 0-0 to 1-1 for positive items and 0-1 to 1-1 for negative items).

EPDS = Edinburgh Postnatal Depression Scale; EPDS-3A = Edinburgh Postnatal Depression Scale–Anxiety Subscale; GAD = generalized anxiety disorder; GHQ = General Health Questionnaire; C-GHQ = chronicity and the GHQ; HADS = Hospital Anxiety and Depression Scale; K-10 = Kessler-10; PTSD = posttraumatic stress disorder; STAI = State-Trait Anxiety Inventory

— = nonreported

**Table 2:** Demographic and clinical characteristics of the study sample (*n* = 240)

Characteristic	Mean	SD
Age, years	30.5	5.7
	*n*	%

Marital status		
Single	52	21.7
Married	153	63.8
Common law	29	12.1
Divorced	0	0
Separated	3	1.3
Other	2	0.8
Not reported	1	0.4
Education		
Incomplete high school	21	8.8
High school	23	9.6
College degree	37	15.4
≥Bachelor’s degree	62	25.8
Not reported	97	40.4
Pregnant status		
Pregnant	155	64.6
Postpartum	85	35.4
Primary psychiatric diagnosis		
Mood disorders	124	51.7
MDD or MDE	108	45.0
Bipolar disorder (I, II, NOS)	11	4.6
Others	5	2.1
Anxiety disorders	49	20.4
GAD	35	14.6
OCD	4	1.7
Social anxiety disorder	5	2.1
PD (with or without AG)	5	2.1
Adjustment disorders	19	7.9
Substance or alcohol use disorders	2	0.8
Other psychiatric diagnosis	15	6.3
No Axis I or II disorder	31	12.9

AG = agoraphobia; GAD = generalized anxiety disorder; MDD = major depressive disorder; MDE = major depressive episodes; NOS = not otherwise specified; OCD = obsessive–compulsive disorder; PD = panic disorder

## Abbreviations

AUC: area under the curve
DSM: Diagnostic and Statistical Manual of Mental Disorders
EPDS: Edinburgh Postnatal Depression Scale
EPDS-3A: EPDS–Anxiety Subscale
GAD: generalized anxiety disorder
GAD-7: GAD 7-item Scale
GHQ: General Health Questionnaire
HADS: Hospital Anxiety and Depression Scale
K-10: Kessler-10
MDD: major depressive disorder
MGMQ: Matthey Generic Mood Question
NPV: negative predictive value
PD: panic disorder
PPV: positive predictive value
STAI: State-Trait Anxiety Inventory
WHCC: Women’s Health Concerns Clinic
